# Characterization of BRCA2 R3052Q variant in mice supports its functional impact as a low-risk variant

**DOI:** 10.1038/s41419-023-06289-8

**Published:** 2023-11-18

**Authors:** Arun Prakash Mishra, Suzanne Hartford, Rajani Kant Chittela, Sounak Sahu, Suhas S. Kharat, Lucia Alvaro-Aranda, Aida Contreras-Perez, Teresa Sullivan, Betty K. Martin, Mary Albaugh, Eileen Southon, Sandra Burkett, Baktiar Karim, Aura Carreira, Lino Tessarollo, Shyam K. Sharan

**Affiliations:** 1 Mouse Cancer Genetics Program, Center for Cancer Research, National Cancer Institute, National Institutes of Health, Frederick, MD USA; 2 Genome Instability and Cancer Predisposition Lab, Department of Genome Dynamics and Function, Centro de Biologia Molecular Severo Ochoa (CBMSO, CSIC-UAM), Madrid, Spain; 3 Laboratory Animal Sciences Program, Frederick National Laboratory for Cancer Research, Frederick, MD USA; 4 Molecular Histotechnology Laboratory, Frederick National Laboratory for Cancer Research, Frederick, MD USA; 5 Present Address: Regeneron Pharmaceuticals, Inc., Tarrytown, NY USA; 6 Present Address: Applied Genomics Section, Bhabha Atomic Research Center, Trombay, Mumbai, 400085 India; 7 Present Address: Department of Biochemistry, Vanderbilt University, Nashville, TN USA

**Keywords:** Cancer genetics, Disease model

## Abstract

Pathogenic variants in BRCA2 are known to significantly increase the lifetime risk of developing breast and ovarian cancers. Sequencing-based genetic testing has resulted in the identification of thousands of BRCA2 variants that are considered to be variants of uncertain significance (VUS) because the disease risk associated with them is unknown. One such variant is p.Arg3052Gln, which has conflicting interpretations of pathogenicity in the ClinVar variant database. Arginine at position 3052 in BRCA2 plays an important role in stabilizing its C-terminal DNA binding domain. We have generated a knock-in mouse model expressing this variant to examine its role on growth and survival in vivo. Homozygous as well as hemizygous mutant mice are viable, fertile and exhibit no overt phenotype. While we did not observe any hematopoietic defects in adults, we did observe a marked reduction in the in vitro proliferative ability of fetal liver cells that were also hypersensitive to PARP inhibitor, olaparib. In vitro studies performed on embryonic and adult fibroblasts derived from the mutant mice showed significant reduction in radiation induced RAD51 foci formation as well as increased genomic instability after mitomycin C treatment. We observed mis-localization of a fraction of R3052Q BRCA2 protein to the cytoplasm which may explain the observed in vitro phenotypes. Our findings suggest that BRCA2 R3052Q should be considered as a hypomorphic variant.

## Introduction

BRCA2 is a well-known tumor suppressor. Some mutations in *BRCA2* are associated with increased risk of breast and ovarian cancer as well as other cancers such as pancreatic and prostate cancer [1]. Sequencing-based genetic testing is now being used to identify mutation carriers. Over 13,000 *BRCA2* variants have been reported in the ClinVar database, of which ~6000 are variants that result in a single amino acid change [2]. Due to limited epidemiological data, functional significance of most variants remains unknown and they are considered to be variants of uncertain significance (VUS) [2]. Although VUSs have been identified in all exons of *BRCA2*, most known pathogenic variants are found in exons that encode the Partner and Localizer of BRCA2 (PALB2) binding domain (exon 3) and the C-terminal DNA binding domain of BRCA2 encoded by exons 15–26 [3, 4]. Loss of heterozygosity in *BRCA2* mutation carriers results in an increase in genomic instability [2]. This results in a cellular environment that is conducive to accumulation of additional mutations and tumor development [5, 6].

BRCA2 plays a key role in repairing DNA double-strand breaks (DSBs) by homologous recombination (HR) by recruiting RAD51 to the damaged DNA. In the absence of BRCA2, RAD51 foci formation at the DSBs is completely abolished. Most pathogenic *BRCA2* variants are defective in HR, its well-established function. HR deficiency has been reported for variants that affect BRCA2 stability [7, 8], nuclear transport [9, 10], DSS1 binding [11, 12] or RAD51 binding [13]. In addition to HR, BRCA2 is required for the protection of stalled replication forks (RFs) from degradation by MRE11 nuclease [14]. In the absence of BRCA2, MRE11 degrades RFs and contributes to genomic instability.

Given the rapid increase in the detection of BRCA2 VUSs, and the need to determine their clinical significance, large scale epidemiological studies have been undertaken [15]. However, because most VUSs are rare, the statistical power of such studies is not significant enough to functionally classify the variants. In recent years, a number of in silico prediction models and in vitro functional assays have been developed that are being used to determine the impact of variants on protein structure and function [16–18].

In our previous studies, we have used a mouse ES cell based system to examine the effect of BRCA2 VUS on cell viability and their response to DNA damaging agents [19]. Among the variants we have examined using this approach, an arginine to tryptophan change at position 3052 (p.Arg3052Trp or R3052W) confers a severe impact on cell viability [19] and is reported to be defective in HR [20]. Arginine at position 3052 of BRCA2 lies at the junction of two oligonucleotide/oligosaccharide-binding folds, OB2 and OB3, that bind to ssDNA. The presence of tryptophan instead of arginine at this position not only disrupts the DNA binding ability of BRCA2 [21] but also its nuclear localization [10, 22]. Based on these findings, R3052W is considered to be a non-functional variant [20–24]. Unlike R3052W, substitution by glutamine at the same residue (p.Arg3052Gln or R3052Q) leads to milder functional consequences. We found that R3052Q support mES cell viability, however, these mES cells exhibited moderate sensitivity to methyl methane sulfonate (MMS), mitomycin C (MMC) and cisplatin [19].

In addition to functional and structural studies, co-segregation studies in families have supported the classification of R3052W as a pathogenic variant. In one of the families with a history of breast cancer, 7 out of 9 patients harbored the R3052W variant [20]. In contrast, multiple studies have suggested R3052Q to be a neutral variant [23, 24]. However, the epidemiological data are not robust enough for the variant to be classified as neutral in ClinVar. Instead, it is considered to be a “likely benign” variant. The physiological impact and cancer risk of BRCA2 R3052Q variant remains unclear.

Mouse models have proven to be useful in examining the functional impact of variants identified in BRCA2. We have previously examined the impact of p.Gly25Arg, which disrupts the interaction with PALB2 [25], and p.Leu2510Pro, which destabilizes the interaction with DSS1 (encoded by *Deleted in split hand/split foot protein 1*) [11] mutations using mouse models. We describe here the generation and characterization of a knock-in mouse model expressing the BRCA2 R2971Q variant (murine R2971 position is equivalent to R3052 position of human BRCA2). We used mutant mice to examine growth, development and disease progression. The mutant mice were born at expected Mendelian ratios and exhibited no overt phenotype. However, we observed a reduction in the radiation-induced RAD51 foci formation in embryonic and adult fibroblasts generated from mutant mice. These cells also exhibited an increase in genomic instability in response to MMC treatment. Overall, our findings suggest that while R3052Q variant does not have a significant impact on BRCA2 function, it is functionally not equivalent to the wild-type protein. It is a hypomorphic variant that exhibits subtle phenotypic defects when challenged with genotoxic stress.

## Results

### *Brca2*^*R2971Q/R2971Q*^ and *Brca2*^*R2971Q/KO*^ mice have normal growth and fertility

**Fig. 1:**
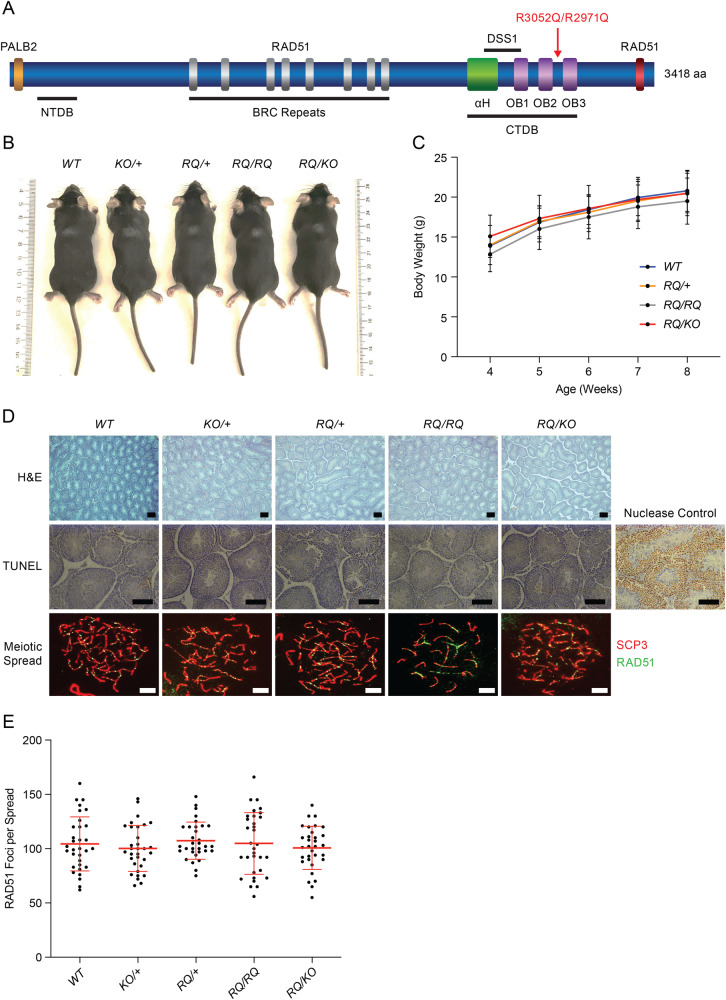
Physiology of *Brca2*^*RQ/RQ*^ and *Brca2*^*RQ/KO*^ mice. **A** Schematic representation of BRCA2 protein showing its functional domains and key binding partners. PALB2 binds to the N-terminal region of BRCA2, RAD51 binds to BRC repeats and the C-terminal region. There is N-terminal DNA binding (NTDB) domain spanning residues 265–341 and a C-terminal DNA binding (CTDB) domain spanning residues 2478–3185. The CTDB domain consists of an α-helical (αH) domain and three oligonucleotides/oligosaccharide bindings folds OB1-3. DSS1 is a small protein that binds to CTDB domain. The R3052Q/R2971Q variant lies at the junction of OB2 and OB3. **B** Representative images of mice of all the genotypes at weaning (21 days). **C** Body weight measurements of mice for 8 weeks post-weaning, mice of all genotypes show normal growth (*n* = 10 animals, one-tailed *t*-test: two-sample assuming unequal variances, error bar—SE of mean). **D** H&E (upper panel), TUNEL (middle panel) and immunofluorescence images of spermatocyte spreads made from testes of 3-week old mice of indicated genotypes (lower panel). H&E staining reveals normal testis morphology in all the genotypes. TUNEL staining suggests lack of abnormal apoptosis in the testes of any genotype. Nuclease control (DNAseI treated) showed TUNEL positive staining. Spermatocyte spreads show chromosomes in late zygotene/pachytene stage labeled with SCP3 (red) and RAD51 (green) antibodies (upper panel scale bar = 200 μm, middle panel scale bar = 100 μm and lower panel scale bar = 5 μm). **E** Quantification of the number of RAD51 foci observed per spermatocyte colocalizing with SCP3 (*n* > 30 spreads per genotype, error bar—SD of mean).

To examine the impact of the BRCA2 R3052Q variant, we generated mice expressing BRCA2 p.R2971Q variant (murine R2971 position is equivalent to R3052 position of human BRCA2). We mutated codon 2971 of *Brca2* in exon 24 from AGG > CAG in mESCs by gene targeting (Fig. 1A and Supplementary Fig. 1A–C). A correctly targeted mESC clone was used to generate *Brca2*^*R2971Q/+*^ mice. These mice are viable and fertile. *Brca2*^*R2971Q/+*^ mice were intercrossed to obtain homozygous mutants (*Brca2*^*R2971Q/R2971Q*^, for simplicity referred to as *Brca2*^*RQ/RQ*^). We also crossed *Brca2*^*RQ/+*^ mice with mice heterozygous for the *Brca2* null allele (*Brca2*^*KO/+*^) to obtain hemizygous *Brca2*^*RQ/KO*^ mice [26]. Both homozygous and hemizygous mice are viable and obtained at expected Mendelian ratios (Table 1). Neither *Brca2*^*RQ/RQ*^ nor *Brca2*^*RQ/KO*^ mice exhibited any gross overt phenotype. Their size and weights were comparable to their control littermates (Fig. 1B, C).

**Table 1:** Observed and expected number of offspring of various genotypes.

*RQ/*+ X *RQ/+*				*RQ/*+ X *KO/+*				
	*+/+*	*RQ/+*	*RQ/RQ*		*+/+*	*RQ/+*	*KO/+*	*RQ/KO*
Observed	44	84	34	Observed	31	50	50	41
Expected	40.5	81	40.5	Expected	43	43	43	43
*χ*^2^ *p* value	0.482			*χ*^2^ *p* value	0.126			

Both *Brca2*^*RQ/RQ*^ and *Brca2*^*RQ/KO*^ mice are fertile and produce offspring with normal litter size (Table 2). We observed no differences in the testes size obtained from adults of different genotypes. Furthermore, histological analysis also confirmed normal morphology and the presence of sperm within the seminiferous tubules (Fig. 1D, upper panel). Also, TUNEL staining on testes sections did not reveal any abnormal apoptotic cells (Fig. 1D, middle panel). Since BRCA2 is required for RAD51 loading on the chromatin during zygotene/pachytene stages of meiosis I [27], we analyzed the spermatocyte spreads from testes of 3-week-old males, for the presence of RAD51. We labeled paired chromosomes with SCP3, a marker of the synaptonemal complex, and RAD51 antibodies. Spreads from *Brca2*^*RQ/RQ*^ and *Brca2*^*RQ/KO*^ mice showed no significant difference in number of RAD51 foci compared to the meiotic spreads from control mice (Fig. 1D, lower panel, E). We also examined the ovaries of *Brca2*^*RQ/RQ*^ and *Brca2*^*RQ/KO*^ mice and found them to be histologically normal and comparable to the mice expressing WT BRCA2 (Supplementary Fig. 2A). All these results confirm that the mutant mice do not have any fertility defects. To assess tissue specific development, we studied terminal end buds (TEBs) formation in mammary gland, as a marker for ductal branching, in 4-week old females [28, 29]. Both *Brca2*^*RQ/RQ*^ and *Brca2*^*RQ/KO*^ mice showed equal number of TEBs compared to their control littermates (Supplementary Fig. 2B, C). Taken together these results reveal that *Brca2*^*RQ*^ homozygous and hemizygous mice are phenotypically indistinguishable from mice expressing wild-type *Brca2* in terms of growth and fertility.

**Table 2:** Average litter size of males when mated with WT females (*n* = number of litters).

Genotype (*n*)	Average litter size
*WT* (21)	7.6
*RQ/+* (21)	6.7
*KO/+* (21)	6.8
*RQ/RQ* (14)	6.6
*RQ/KO* (17)	7.5

### *Brca2*^*RQ/RQ*^ and *Brca2*^*RQ/KO*^ MEFs show reduced radiation-induced RAD51 foci formation

**Fig. 2:**
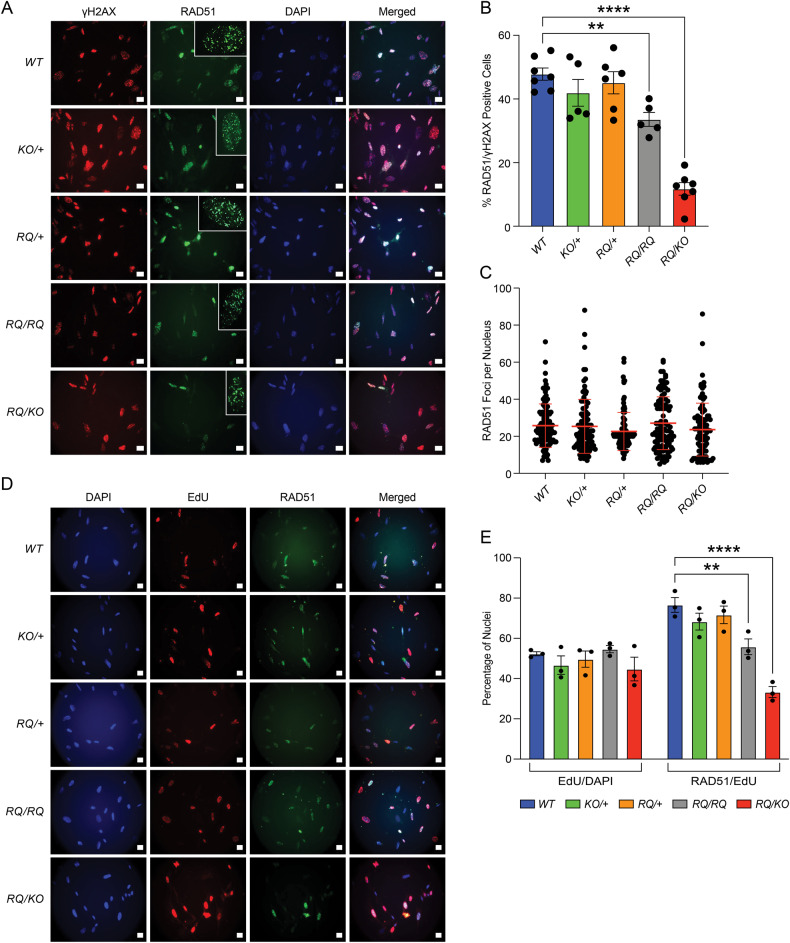
*Brca2*^*R2971Q*^ MEFs are deficient in RAD51 loading after radiation. **A** Representative images of immunofluorescence in MEFs showing RAD51 foci formation after 3 h of 10 Gy IR. Nuclei are marked with DAPI and DSBs with γH2AX. Inset shows number RAD51 foci per cell (scale bar = 20 μm). **B** Quantification of RAD51-positive nuclei per γH2AX positive of different genotypes. *RQ/RQ* and *RQ/KO* MEFs exhibit significantly lower number of RAD51 foci positive nuclei (*n* = 3 biological replicate, ordinary one-way ANOVA, error bar—SE of mean, **p < 0.01, ****p < 0.0001). **C** Number of RAD51 foci per nucleus in each genotype (*n* > 100 nuclei per genotype, error bar—SD of mean). **D** Representative images of EdU and RAD51 labeling in MEFs after 1 h of 10 Gy IR (scale bar = 20 μm). **E** Quantification of EdU and RAD51/EdU-positive nuclei (*n* = 3 biological replicate, two-way ANOVA, error bar—SE of mean, **p < 0.01, ****p < 0.0001).

Arginine at position 2971 (3052 in human BRCA2) is at the junction of oligosaccharide-binding domains, OB2 and OB3, and is predicted to stabilize BRCA2 structure [12, 19]. Substitution of arginine to glutamine is predicted to have a mild impact on the structural integrity and DNA binding ability of BRCA2 [19]. This may reduce its ability to recruit RAD51 during repair of DSBs by HR. To assess the impact of R2971Q variant on RAD51 recruitment, we exposed MEFs, isolated from 13.5 days post coitum (dpc) embryos, of all genotypes to 10 Gy IR (ionizing radiation) and examined for RAD51 foci formation after 3 h. We detected formation of DSB by γH2AX staining, which is a pan-DSB marker, and measured the percentage of γH2AX positive cells containing RAD51 foci. We found 40–55% of *Brca2*^*+/+*^*, Brca2*^*KO/+*^, and *Brca2*^*RQ/+*^ MEFs positive for RAD51 foci. In contrast, the number of RAD51 foci positive cells decreased to 27–35% in *Brca2*^*RQ/RQ*^ or 3–15% in *Brca2*^*RQ/KO*^ MEFs (Fig. 2A, B). A reduced percentage of RAD51 foci positive mutant MEFs suggests that BRCA2 R2971Q mutation reduces RAD51 nucleofilament formation at DSBs. Surprisingly, the total number of RAD51 foci per nucleus was found to be comparable in each genotype (Fig. 2A inset, C). As it is known that RAD51 mediated DSB repair occurs during S and G2 phases of cell cycle [30], reduced number of RAD51 foci positive R2971Q cells could be because of slow growth rate or cells stalling in G1 phase. To analyze this, we EdU-pulse labeled the MEFs of all genotypes for 1 h after 10 Gy IR and probed for RAD51 foci formation to monitor the cells in S-phase. We did not find any difference in the percentage of EdU-positive cells in MEFs of all genotypes (Fig. 2D, E). However, we observed reduced number of RAD51/EdU-positive cells, in both homozygous and hemizygous MEFs (Fig. 2E). These results suggest that the deficiency of RAD51 loading in mutant MEFs is not because of cell cycle stalling.

To assess whether the reduction in RAD51 foci positive cells persisted in adult mice, we isolated fibroblasts from ear punch of adult mice (8–10-week-old) of all genotypes. We exposed them to 10 Gy radiation and examined for RAD51 foci formation. Similar to the defect seen in MEFs, *Brca2*^*RQ/KO*^ adult fibroblasts displayed reduced percentage of RAD51/γH2AX positive cells; whereas this percentage in *Brca2*^*RQ/RQ*^ cells were similar to that observed in *Brca2*^*KO/+*^*, Brca2*^*RQ/+*^ or WT cells (Supplementary Fig. 3A, B). As found for the MEFs, the overall number of RAD51 foci per nucleus did not change among genotypes (Supplementary Fig. 3A, C).

Given the reduced number of RAD51 foci positive mutant cells, we examined the effect of BRCA2 R2971Q mutation on the protection of stalled replication forks (RF). We performed DNA fiber assay using *Brca1del11* (*Brca1*^*Δ11/Δ11*^) MEF as control for unprotected stalled RFs [31]. This assay was performed by sequentially labeling RFs with CldU (red) and IdU (green) for 30 min each followed by stalling the forks by hydroxyurea (HU) treatment for 3 h [14]. We found that the ratio of IdU to CldU track lengths was close to one in MEFs of all genotypes except *Brca1*^*Δ11/Δ11*^ (Supplementary Fig. 3D) suggesting that *Brca2*^*RQ/RQ*^ and *Brca2*^*RQ/KO*^ MEFs are proficient in protecting nucleotide-depleted stalled RFs. These results suggest that although BRCA2 R2971Q mutation manifests reduced number of IR-induced RAD51 foci positive cells but does not affect the stalled RF protection function of BRCA2.

### *Brca2*^*RQ/KO*^ fetal livers show reduced colony-forming units and are sensitive to genotoxic stress

**Fig. 3:**
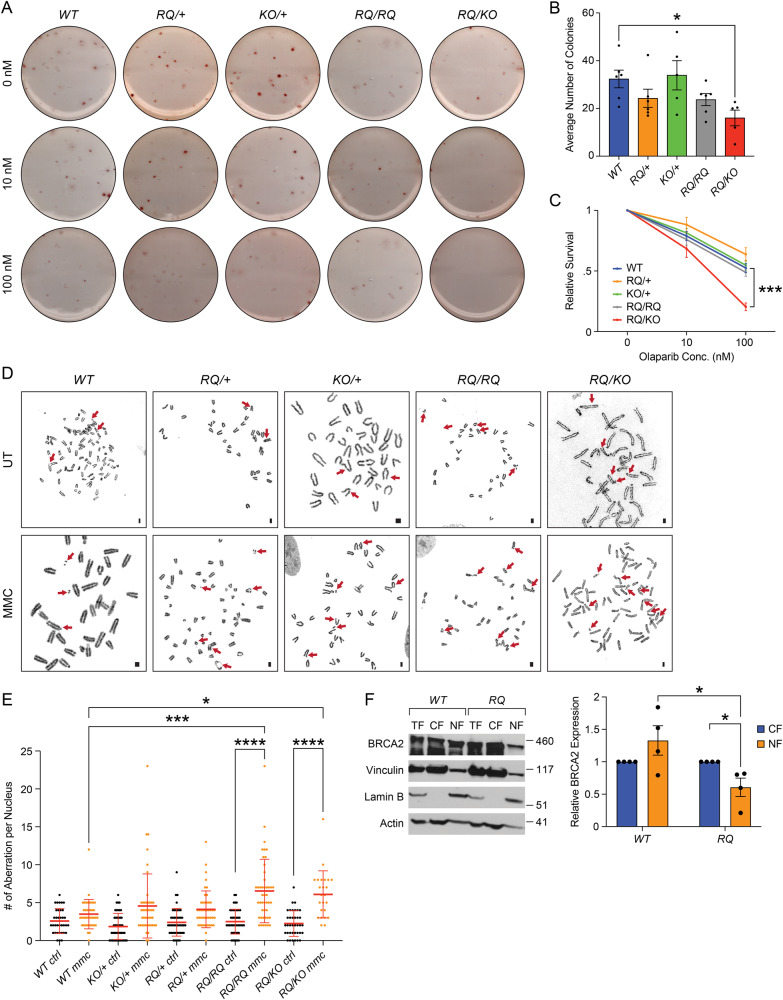
*Brca2*^*R2971Q*^ mutation makes cells sensitive to DNA damaging drugs. **A** Representative images of colony-forming assay of hematopoietic progenitor cells from fetal liver isolated from 16.5 dpc embryos of all genotypes with different concentration of olaparib. **B** Quantification of CFUs in untreated condition (*n* = 3 biological replicates, *p < 0.05, ordinary one-way ANOVA, error bar—SE of mean). **C** Relative number of colonies formed from the fetal liver cells when exposed to increasing concentration of olaparib (*n* = 3 biological replicates, one-tailed *t*-test: two-sample assuming unequal variances, error bar—SE of mean, ***p < 0.001). **D** Chromosomal aberrations in MEFs of indicated genotype in untreated (ctrl) and 100 nM MMC treated conditions (scale bar = 10 μm). Aberrations are marked with arrows. **E** Quantification of chromosomal aberrations in MEFs shown in (**D**). *RQ/RQ* and *RQ/KO* MEFs exhibit increased number of chromosomal aberrations after MMC treatment as compared to WT (*n* > 24 spreads per genotype per treatment, two-way ANOVA, error bar—SD of mean, *p < 0.05, ***p < 0.001, ****p < 0.0001). **F** Western blot performed on whole cell lysate or total fraction (TF), cytoplasmic fraction (CF) and nuclear fraction (NF) of mES cells expressing WT and R3052Q BRCA2. Vinculin, Lamin B and Actin are used as CF, NF and TF loading controls, respectively. Graphical representation of amount of BRCA2 protein detected in the western blot normalized to Actin loading control (*n* = 4 technical replicates (2 biological), *p < 0.05, Student’s *t* test, error bar—SE of mean).

Given the fibroblasts from mutant genotypes exhibit deficiency in IR-induced RAD51 foci formation, we used fetal liver cells to examine their sensitivity of hematopoietic progenitors to PARP inhibitors (olaparib). Cells from freshly isolated fetal liver, at 16.5 dpc, were dissociated and plated to allow formation of colonies. Although, *Brca2*^*RQ/RQ*^ fetal liver cells produced similar number of colony-forming units (CFUs) as the controls (Fig. 3A, B), the *Brca2*^*RQ/KO*^ fetal liver cells displayed significantly fewer CFUs in untreated condition (Fig. 3A, B) also they were found to be hypersensitive to olaparib treatment (Fig. 3C). These results suggest that R2971Q hemizygosity confers reduced proliferation capacity and hypersensitivity to PARPi in fetal liver cells, in line with the results observed in the MEFs which showed a significant reduction in RAD51-positive cells.

Based on the significant defect in *Brca2*^*RQ/KO*^ fetal liver cells, we examined whether there is any defect in the hematopoietic cells in adults. We performed complete peripheral blood analysis of 8-week-old mice and did not observe any defect there. All the blood cells including red blood cells and white blood cell in *Brca2*^*RQ/RQ*^ and *Brca2*^*RQ/KO*^ mice were within the normal range and comparable to the control groups (Supplementary Fig. 4A). This suggests that although we observed a defect in the proliferative ability of hematopoietic progenitors in vitro, under physiological conditions there was no apparent hematological defect.

To better understand the effect of BRCA2 R2971Q mutation on genomic integrity, MEFs of all genotypes were examined for chromosomal aberrations with/out MMC treatment. *Brca2*^*RQ/RQ*^ and *Brca2*^*RQ/KO*^ MEFs did not show higher chromosomal aberrations in untreated conditions. As expected, MMC treatment increased the total number of chromosomal aberrations in all the cell types. Importantly, this increase was significantly exacerbated in *Brca2*^*RQ/RQ*^ and *Brca2*^*RQ/KO*^ MEFs (Fig. 3D, E). These results suggest that the BRCA2 R2971Q mutation is proficient in maintaining the genomic integrity under physiological conditions, however, when challenged with genotoxic stress, both *Brca2*^*RQ/RQ*^ and *Brca2*^*RQ/KO*^ MEFs exhibit genomic instability.

Mutations in the C-terminal DNA binding domain of BRCA2 have been reported to affect its nuclear localization [10, 22]. Because of lack of suitable antibodies to detect the endogenous murine BRCA2 in MEFs, we performed western blot analysis of protein isolated from mES cells harboring human WT and *R3052Q BRCA2* gene cloned in a BAC [19]. WT and R3052Q BRCA2 were detected in both cytoplasmic and nuclear fraction; however, BRCA2 R3052Q displayed lower relative protein levels in the nuclear fraction as compared to that observed for WT (Fig. 3F). This mis-localization of BRCA2 R3052Q in the cytoplasmic fraction might be due to a reduced structural stability of the interface between OB2-OB3 caused by arginine to glutamine substitution, as also suggested for R3052W [32].

### *Brca2*^*RQ/RQ*^ and *Brca2*^*RQ/KO*^ animals have normal life span and *Brca2*^*RQ/KO*^ animals are mildly sensitive to radiation

**Fig. 4:**
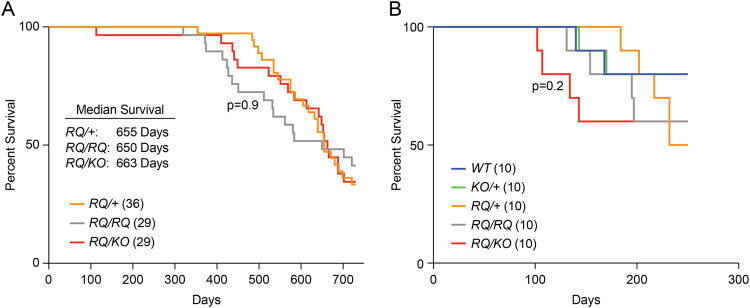
*Brca2*^*R2971Q*^ mutant mice have normal survival and *Brca2*^*RQ/KO*^ mice are mildly sensitive to radiation. **A** Kaplan–Meier survival curve for 750 days of mice of indicated genotypes (*n* for each genotype is mentioned in the parentheses, Log-rank Mantel–Cox test). **B** Kaplan–Meier survival curve of mice after 7 Gy IR, mice monitored for 250 days post radiation. RQ/KO mice started to die early showing mild sensitivity to radiation as compared to all other genotypes (*n* = 10 for each genotype, Log-rank Mantel–Cox test).

To examine the impact of BRCA2 R2971Q variant on tumor predisposition, we generated a cohort of *Brca2*^*RQ/+*^, *Brca2*^*RQ/RQ*^ and *Brca2*^*RQ/KO*^ animals and monitored their tumor-free survival for 750 days. We did not see any significant difference in tumor-free survival in any genotypic group (Fig. 4A). When we examined the tumors developed in these mice, there was no significant difference in the tumor spectrum between different genotypic groups. The most common tumor type observed was hematopoietic neoplasm in all groups (Table 3). These results suggest that although fibroblasts and fetal liver cells exhibit altered phenotype, BRCA2 R2971Q variants does not significantly predispose *Brca2*^*RQ/KO*^ mice to tumorigenesis.

**Table 3:** Tumor incidence and spectrum of *Brca2*^*RQ/+*^, *Brca2*^*RQ/RQ*^ and *Brca2*^*RQ/KO*^ mice.

	*RQ/+*		*RQ/RQ*		*RQ/KO*	
# of mice	36		29		29	
# of mice with all neoplasm	24 (66.67%)		20 (69%)		18 (62%)	
	Females	Males	Females	Males	Females	Males
# of mice	17	19	20	9	16	13
Hematopoietic neoplasm	16	6	13	4	9	3
Benign neoplasm	3	1	4	1	6	3
Malignant neoplasm	1	5	8	1	3	3
Animals with multiple neoplasms	5	2	7	1	5	2

The reduction in RAD51 recruitment at the DSBs observed in *Brca2*^*RQ/RQ*^ and *Brca2*^*RQ/KO*^ fibroblasts and sensitivity of fetal liver cells to olaparib suggests a defect in HR. Therefore, we hypothesized that these mice may be sensitive to γ-irradiation. To test this, we exposed mice of all genotypes to 7 Gy of sublethal γ-radiation and monitored their survival. *Brca2*^*KO/+*^*, Brca2*^*RQ/+*^ or *Brca2*^*RQ/RQ*^ mice showed no survival defect compared to the *Brca2*^*+/+*^ littermates. In contrast, the *Brca2*^*RQ/KO*^ mice were mildly sensitive to radiation and started to die early (although not statistically significant, *p* = 0.2) (Fig. 4B).

## Discussion

Inheritance of a cancer causing pathogenic BRCA2 variant significantly increases the disease risk in mutation carriers. Currently, the risk associated with the majority of BRCA2 variants, especially those that result in a single amino acid substitution, remains unknown. Given the clinical implications, it is important to understand the functional impact of BRCA2 VUSs. Classification of BRCA2 VUS is a rapidly evolving field; pathogenicity of variants is determined based on all the evidence that are available. Often variants are reclassified when a new evidence becomes available that does not support previous classification. In absence of strong data/evidence for clinical classification a certain variant is considered to be VUS, restricting the counseling of individuals who inherit a VUS.

The present study is focused on understanding the physiological significance of BRCA2 R3052Q variant with conflicting interpretations of pathogenicity in ClinVar. This interpretation is based on epidemiological studies examining disease occurrence in mutation carriers. This variant has been evaluated by multiple in vitro functional assays and in most but not all, it was indistinguishable from WT and neutral variants [23, 24]. One study determined BRCA2 R3052Q mutation to be sensitive to cisplatin and 50% reduction in GFP based HR assay [33]. A sequencing-based study performed on Japanese patients classified it as pathogenic variant [34]. Another report compiled results from various researches integrating variant population-frequency, classified it as likely benign [35]. The current study, along with our previous study on mES cells [19], indicate that BRCA2 R3052Q variant confers olaparib sensitivity, reduced IR-induced RAD51 foci formation and increased genomic instability after MMC treatment suggesting it to be a hypomorphic variant.

Mice expressing BRCA2 R2971Q did not exhibit any developmental or fertility defects and had normal longevity. Mutant animals were also not significantly sensitive to sublethal dose of IR. These phenotypes suggest R2971Q variant to be functionally neutral. However, we observed mutant MEFs exhibiting increased chromosomal aberrations after MMC treatment. Also, the hemizygous mutant (*Brca2*^*RQ/KO*^) fetal liver cells displayed fewer CFUs and were hypersensitive toward olaparib. These results are consistent with the observed deficiency in radiation-induced RAD51 foci formation in mutant fibroblasts. Surprisingly, the mutant nuclei which showed RAD51 foci, had similar number of foci in them as compared to control cells hinting that there is a cell-to-cell variation. Taken together, these results suggest that the mice expressing this variant can cope with DNA damage generated under physiological conditions but the mutant cells exhibit a defective phenotype when cultured in vitro or challenged with genotoxic stress.

It is known that mutations in the C-terminus of BRCA2 result in defective oligomerization and mis-localization of the protein in the cytosol [10]. Although this may explain a part of the phenotype we observe, the fact that some amount of protein locates in the nucleus and the RAD51 foci are not completely abolished, suggest that other aspects of the protein, for example its reduced stability in the nucleus, might be at play. Similarly, a fraction of BRCA2 R3052W protein, a deleterious variant affecting the same residue as R3052Q, locates in the nucleus and is recruited to the RFs showing functional protein at HU-stalled RFs [36]. These data suggest that both R3052W and R3052Q share sensitivity to PARPi and HR deficiency to different degrees. It is possible that due to cellular heterogeneity, there may be a cell-to-cell variability in the amount of nuclear vs. cytoplasmic protein, accounting for the differences observed between cell lines and in the in vivo experiments. It is possible that cells that have the protein below a certain threshold, fail to mediate HR by RAD51 and exhibit a defective phenotype when challenged with genotoxic stress (IR, olaparib or MMC). This may also contribute to slight (albeit not statistically significant) increase in the lethality of mice after radiation. Overall, our functional evaluation provides strong data to classify BRCA2 R3052Q to be a hypomorphic variant.

## Methods

### Generation of *Brca2*^*R2971Q*^ knock-in mice

*Brca2*^*R2971Q*^ knock-in mice were generated by gene targeting approach in mouse V6.4 ES cells [37]. To generate the targeting construct, two nucleotides (AGG to CAG) of codon 2971 encoded by exon 24 of *Brca2* cloned in a bacterial artificial chromosome (BAC) containing full length *Brca2* (RPCI22-421-23A) were changed by recombineering using a *galK-*based selection-counterselection method [38, 39]. We inserted a *loxP*-PGK-Tn5-*neomycin*-bp(A)-*loxP* cassette along with an *Sph*I restriction site in intron 24 by recombineering. We then retrieved a 9 kb genomic region of *Brca2* from the BAC into pBSK(+) plasmid by gap repair. pBSK(+) plasmid contained the *Thymidine Kinase* (TK) gene under the control of the MC1 promoter, which was used for negative selection [40]. The targeting construct was linearized with *Not*I restriction enzyme and electroporated into V6.4 ES cell line as described previously [37]. G418 and FIAU resistant ES cell clones were screened by Southern analysis using *Sph*I and *Dra*III restriction enzyme digested genomic DNA using 5´ and 3´ probes (Supplementary Fig. 1). Two correctly targeted ES cell clones were injected into C57BL/6 mice blastocysts. Some of the chimeras obtained transmitted the targeted allele in the germ line and *Brca2*^*R2971Q-Neo/+*^ pups were obtained. *Brca2*^*R2971Q-Neo/+*^ mice were crossed to β-actin-Cre mice to delete the *Neo* cassette and obtain *Brca2*^*R2971Q/+*^ (*Brca2*^*RQ/+*^) mice [41]. *Brca2*^*RQ/+*^ mice were intercrossed to generate *Brca2*^*RQ/RQ*^ mice. We backcrossed *Brca2*^*RQ/+*^ mice (mixed C57BL/6 and 129/SvEv background) to C57BL/6 mice for 10 generation to obtain *Brca2*^*RQ/+*^ mice on a pure background. We also generated hemizygous mice (*Brca2*^*RQ/KO*^), by crossing *Brca2*^*RQ/RQ*^ mice with *Brca2*^*KO/+*^ mice carrying a *Brca2* knock-out (*KO*) allele [42].

Mice were housed and bred following the recommendations of the Guide for the Care and Use of Laboratory Animals (The National Academies Press; 8th edition). The study protocol was approved by the Animal Care and Usage Committee (ACUC) of NCI-Frederick (Animal Study# 21-471). Mice were maintained at a 12 h light/dark cycle. Rooms were maintained at temperatures in the range of 20–27 °C and 30 to 70% relative-humidity. All animal studies were performed in compliance with ARRIVE (Animal Research: Reporting In Vivo Experiments) guidelines (https://arriveguidelines.org/arrive-guidelines).

### Genotyping

Genomic DNA was extracted from tail clips by incubating them in lysis buffer (100 mM Tris pH8.8, 5 mM EDTA, 200 mM NaCl, 100 mg/ml Proteinase K) at 55 °C overnight. Genotyping was performed by PCR using gDNA with QuickTaq master mix (DTM-101, Toboyo) using primers listed in Supplementary Table 1.

### Histology

Overnight fixed tissues (10% formalin) were paraffin-embedded, sectioned (5 µm) and H&E (hematoxylin and eosin) stained. TUNEL staining on sections were performed following the manufacturer’s directions of the In Situ Cell Death Detection Kit (Roche 11684817910). For aging studies, the histopathology analyses were performed blindly.

### Meiotic chromosome analysis

The testes were excised from young males (3-week old) and rinsed in PBS and followed the protocol as explained earlier [43]. Briefly, the excised testes were transferred to hypo-extraction buffer (50 mM sucrose, 17 mM Tri sodium citrate, 5 mM EDTA, 0.5 mM DTT, 0.1 mM PMSF, 30 mM Tris pH8.2). The tunica was peeled off gently and the tubules were teased using forceps and incubated in hypo-extraction buffer for 30–60 min on ice. On a clean slide, 25 μl of 100 mM sucrose solution bubble was placed and one fourth portion of incubated testis section from the previous step was added. Using a pipette, the tissue was minced up and down several times so as to get a cloudy slurry. On a separate labeled slide, kept in fixative solution (1% paraformaldehyde, 10% Triton X-100 in PBS, pH9.2) in a coplin jar, 20 μl of the cloudy slurry from the previous step is spread evenly. The slides were kept in a humid chamber for drying. After drying the slides were stored at −20 °C. For staining, the slides were briefly rinsed with PBS and then incubate with primary antibodies: rabbit anti-RAD51 (1:250, Millipore PC130); mouse anti-SCP3 (1:500 Santa Cruz sc-74568) diluted in antibody dilution buffer and followed the protocol as for immunofluorescence as discussed below.

### Carmine Alum staining of mammary gland

Mammary glands from 4-weeks old female mice were fixed in Carnoy’s solution (6:3:1 of ethanol, chloroform and acetic acid). The glands were rehydrated in decreasing grades of ethanol and stained with Carmine Alum (C1022, Sigma) overnight. Subsequently the glands were dehydrated using increasing grades of ethanol and left for tissue clearing for 2–3 days in xylene substitute.

### Generation of mouse embryonic fibroblasts (MEFs)

Embryos from timed mating (13.5 dpc) of specific genotypes were obtained. Specific embryos were chopped finely in 0.5% trypsin (15400-054, Gibco) and incubated at 37 °C for 30 min. The slurry was pipetted multiple times to break clumps and plated on gelatinized 100 mm culture plates in DMEM + 10% FBS. After confluency small aliquots of these P0 (passage zero) cells were frozen in liquid nitrogen.

### Generation of adult fibroblasts (AFs)

Five to ten mm ear punch of animals of all genotypes were obtained and briefly washed in 70% ethanol followed by three washes with sterile Hank’s balanced salt solution (HBSS) for 5 min each. The ear punches were finely chopped in collagenase (C7657, Sigma; 2000IU/ml in HBSS) and incubated for 3 h at 37 °C. After centrifugation at 3000 × *g* for 5 min supernatant was removed and pellet was incubated in 0.5% trypsin (15400-054, Gibco) at 37 °C for 30 min. Digested slurries were plated on gelatinized 100 mm culture plates in DMEM + 10% FBS. After confluency small aliquots of these P0 cells were frozen in liquid nitrogen for future use.

### Immunofluorescence

MEFs and AFs were counted (50,000 cells per coverslip) and seeded on poly-D-Lysine coverslips (Neuvitro GG-12). Cells were irradiated (10 Gy) followed by 3 h of recovery. Cells were incubated in hypotonic solution (5 mM MgCl_2_, 85.5 mM NaCl, pH 7) followed by fixing (4% Paraformaldehyde, 10% SDS in PBS) for 10 min each at room temperature. Cells were incubated with primary antibodies: γH2AX (1:500, Millipore JBW301) and RAD51 (1:250, Millipore PC130) diluted in antibody dilution buffer (5% goat serum, 1% BSA, 0.3% Triton X-100 in PBS) overnight at 4 °C. Following morning, cells were washed three times with PBS containing 0.2% Triton X-100 (PBST) and incubated with secondary antibodies at 37 °C for 1 h: Alexa-fluor anti-mouse 594 (1:500, Invitrogen A11005) and anti-rabbit 488 (1:500, Invitrogen A11034) diluted in PBS. Cells were washed 2 times with PBST and stained for DAPI (1:50,000, Sigma 11190301) for 1 min and again washed 2 times with PBST. Coverslips were mounted on labeled slides using anti-fade mount (Invitrogen P36930).

### EdU and RAD51 labeling in MEFs

MEFs of all the genotypes were counted (50,000 cells per coverslips) and seeded on poly-D-Lysine coverslips (Neuvitro GG-12). The cells were radiated (10 Gy) and were grown for 1 h in 10 μM EdU containing media. After 1 h the cells were permeabilized and fixed as explained above. The MEFs were subjected to in situ click reaction. Briefly, fixed MEFs were washed twice with PBS followed by incubation in click reaction solution (10 mM sodium ascorbate, 10 μM biotin azide, 2 mM CuSO_4_ dissolved in PBS) for 2 h at room temperature in dark. After the click reaction, MEFs were washed twice with PBST and incubated overnight at 4 °C with anti-biotin antibody (Bethyl-A150-109A, 1:500) and rabbit anti-RAD51 (1:250, Millipore PC130) diluted in antibody dilution buffer. Rest of the steps were similar to regular immunofluorescence as described above.

### DNA fiber assay

For DNA fiber analysis, 2 × 10^5^ MEFs were plated in a 12 well plate. Thirty-minute pulses each of CldU (8 μg/ml) and IdU (90 μg/ml) were given in prewarmed DMEM + 10% FBS. Following the pulses of thymidine analogs, the cells were washed with PBS and incubated for 3 h 4 mM HU (hydroxyurea, Sigma H8627) in DMEM + 10% FBS. After the HU treatment cells were trypsinized and resuspended in PBS. Cell lysis was performed on slide by adding cell 5 μl suspension to 15 μl lysis buffer 0.2 M Tris pH 7.5, 50 mM EDTA and 0.5% SDS) and incubating for 10 min at room temperature. The slides were tilted against a support for fibers to spread and dry. Fibers were fixed overnight using methanol: acetic acid (3:1) mixture at 4 °C followed by PBS rehydration and denaturation for 1 h in 2.5 M HCl. The slides were rinsed twice with PBST and blocked for 40 min with 5% BSA in PBST. The fibers were then incubated with primary rat anti-BrdU antibody (Abcam ab6326, 1:500) and mouse anti-BrdU antibody (Becton Dickinson 347580, 1:500) for 2.5 h in the blocking solution. Slides were rinsed with PBST and incubated with secondary antibodies (diluted in blocking solution) for 1 h at room temperature: anti-rat AlexaFluor594 (1:500, Invitrogen A11007) and anti-mouse AlexaFluor488 (1:100, Invitrogen A21202). The slides were washed three times with PBST and were mounted using anti-fade mount (P36930, Invitrogen).

### Fetal liver cells colony-forming assay

Fetal liver cells were harvested from embryos (16.5 dpc) and dissociated in IMDM using a 40 μm cell strainer. In total, 50,000 liver cells were suspended in 1 ml MethoCult (M3231, Stem Cell Technologies) with growth factors (100 ng/ml muSCF,100 ng/ml huTpo, 100 ng/ml huFlt3L, 30 ng/ml muIL3, 50 ng/ml muIL6, 10% FBS all from PeproTech) and plated in 6-well plates. Fetal liver cells were allowed to grow until visible colonies started to appear (7–10 days). Colonies were stained with Iodonitrotetrazolium chloride solution (200 μl/well, 1 mg/ml in PBS; Sigma I10406) overnight and counted.

### Cytogenetic analyses

MEFs of all the genotypes (10^5^ cells, grown in 6-well plate) were either untreated or treated with MMC for 24 h and released in normal media for 6 h. Cells were arrested in metaphase by using colcemid (10 µg/ml, 15210-016, KaryoMAX) diluted in regular media for 12 h at 37 °C. The cells were harvested and fixed in methanol: acetic acid (3:1) solution. Metaphase spreads were stained with Giemsa. Quantification of chromosomal aberrations was done blindly.

### Western blotting

mESC harboring WT and R3052Q mutant BRCA2 were cultured and harvested. The cell pellet was used to isolate total, cytoplasmic and nuclear protein fraction. For total protein fraction the cells were lysed in lysis buffer (20 mM HEPES, 100 mM NaCl, 1 mM EDTA, 1 mM EGTA, 0.1% Triton X-100, 1 mM DTT) for 2 h at 4 °C. Cytoplasmic protein fractionation was performed using a commercially available kit (78833, Thermo Scientific). For nuclear protein extraction, we used the same lysis buffer (as for total extract). The lysates were heated at 95 °C for 10 min with Laemmli buffer. The lysates are cooled down and loaded on a 3–8% gradient SDS PAGE gel (EA0378BOX, Invitrogen). Transfer to nitrocellulose membrane (84-876, Genesee Scientific) was done overnight at 55 volts. The transferred membrane was cut according to the protein ladder marks (LC5699, Thermo Scientific) and blocked for 2 h in 5% BSA in TBST. Primary antibodies: BRCA2 (A303–434A; Bethyl Laboratories, 1:2000), Vinculin (sc-73614, Santa Cruz, 1:50,000), actin (I-19: sc-1616; Santa Cruz Biotechnology, 1:10,000) and Lamin B1 (12987-1-AP, Proteintech 1:50,000); diluted in blocking solution were used for overnight incubation of the membranes at 4 °C. After washing three times with TBST the membranes were incubated at room temperatures with respective secondary antibodies (HRP conjugated) diluted in 3% BSA in TBST. The blots were visualized using luminol (RPN2236, Amersham) and X-ray films. The densitometric strength of the bands observed were determined using Fiji.

### Animal survival study post IR

Animals from each genotype (6–8 weeks old) were given 7 Gy of γ-irradiation from a Cs source. Mice were closely monitored for 250 days after irradiation, any animal showing signs of distress including weight loss was euthanized following ACUC guidelines.

### Statistical analysis

Statistical analyses were performed using GraphPad Prism version 6.0. Statistical tests, *p* values and error bars are reported for all experiments in the figure legends.

### Supplementary information

Supplemental Figures 1-4 and Supplementary Table 1

Reproducibility checklist

## Data Availability

All the required data is provided with the manuscript in terms of figures, tables, supplementary figures and original blots. Additional information will be provided upon request.
