# Supplementary material for: Characterization of BRCA2 R3052Q variant in mice supports its functional impact as a low-risk variant
Source: Cell Death Dis. 2023 Nov 18;14(11):753. doi: 10.1038/s41419-023-06289-8 (PMC10657400; doi:10.1038/s41419-023-06289-8)
Supplement: Supplementary file 1 — Supplemental Figures 1-4 and Supplementary Table 1 [file 41419_2023_6289_MOESM1_ESM.pdf]

Supplementary Figure 1: Generation of *Brca2^{R2971Q}* knock-in mice.

A) A schematic representation of targeting strategy to generate *Brca2^{R2971Q}* knock-in allele in mESC. Open boxes with numbers represent the exons with appropriate exon numbers and lines connecting the boxes represent the introns. Not drawn to scale. A targeting vector containing 4.6kb 5′ homology and 4.4kb 3′ homology arms; a *loxP-PGK-neo-loxP* cassette for positive selection and *Thymidine Kinase* gene *(TK)* for negative selection was used to target the wild type *Brca2* allele (*Brca2^{WT}*). The 5' homology arm contains AGG>CAG mutation in exon 24 that results in arginine to glutamine substitution in codon 2971. Homologous recombination of the targeting vector resulted in generation of *Brca2$^{R2971Q\text{-}Neo}$* allele. Mice heterozygous for *Brca2$^{R2971Q\text{-}Neo}$* allele were crossed with Cre expressing mice to delete the *Neo* cassette leaving behind a single *loxP* site in intron 24 to generate the *Brca2^{R2971Q}* knock-in allele. Relevant restriction sites are indicated, D: *DraIII*; S: *Sph*I. B) We picked G418 resistant colonies and identified the correctly targeted one by Southern analyses of genomic DNA digested with *Sph*I for 3′ end targeting and *DraIII* for 5′ end targeting using probes shown in A (below the *Brca2^{WT}* locus). C) The mutation was confirmed by sequence analysis. D) Schematic representation of the *Brca2* null allele (*Brca2^{Ko}*) showing deletion of 5′ region of exon 11, which is replaced with human *HPRT1* minigene.

Supplementary Figure 1

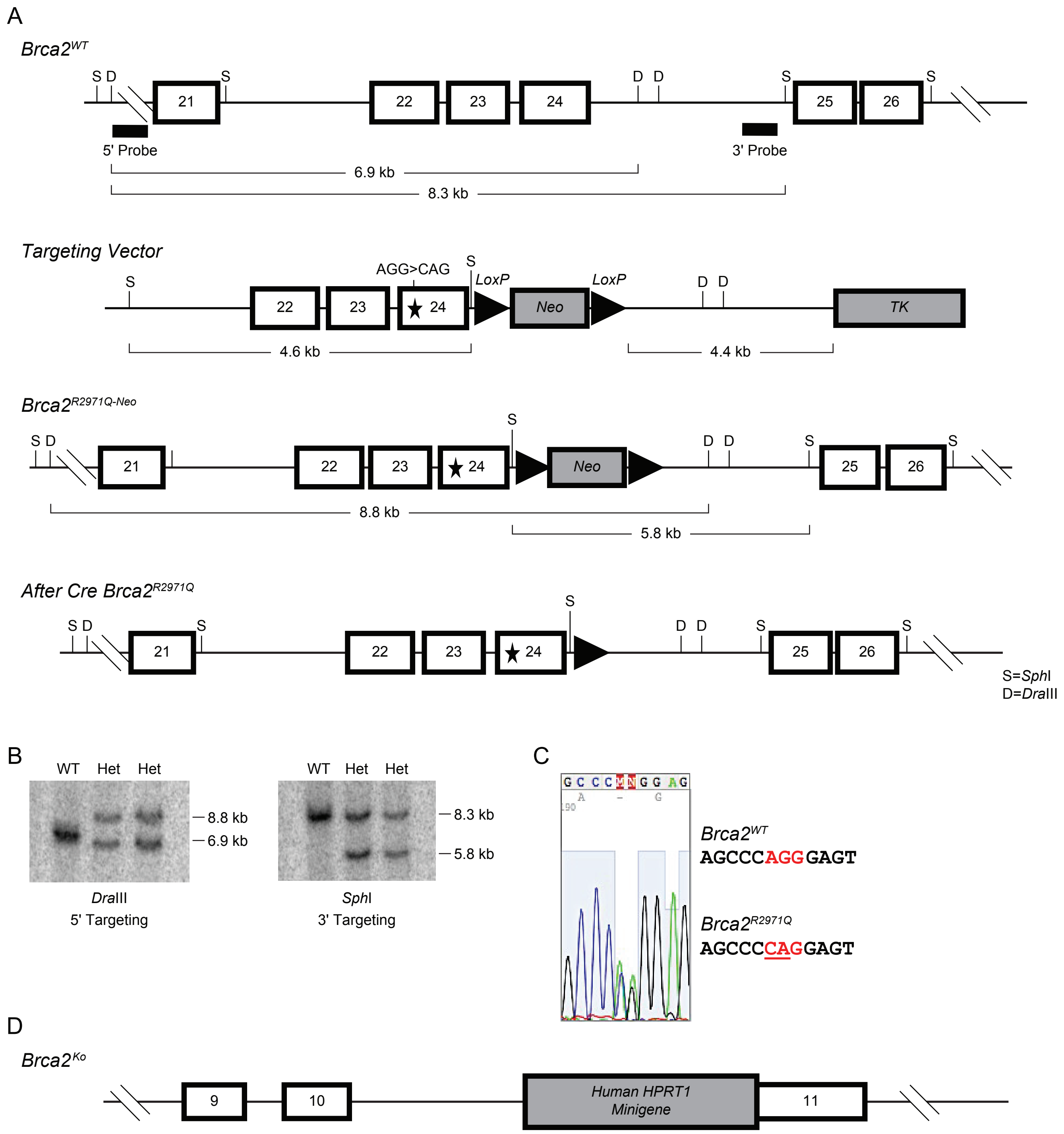

Supplementary Figure 2: Female ovaries and mammary gland .

A) H&E staining of 3-4 weeks old mice ovaries of all the genotypes. Distinct Corpus lutea and developing follicles can be seen in each genotype (n=3 mice per genotype, scale bar=300µm). B) Representative images of carmine alum-stained mammary gland of 4 weeks old females of all genotypes. C) Quantification of number of terminal end buds (TEBs) observed (B) (n=8 glands per genotype, error bar- SE of mean).

Supplementary Figure 2

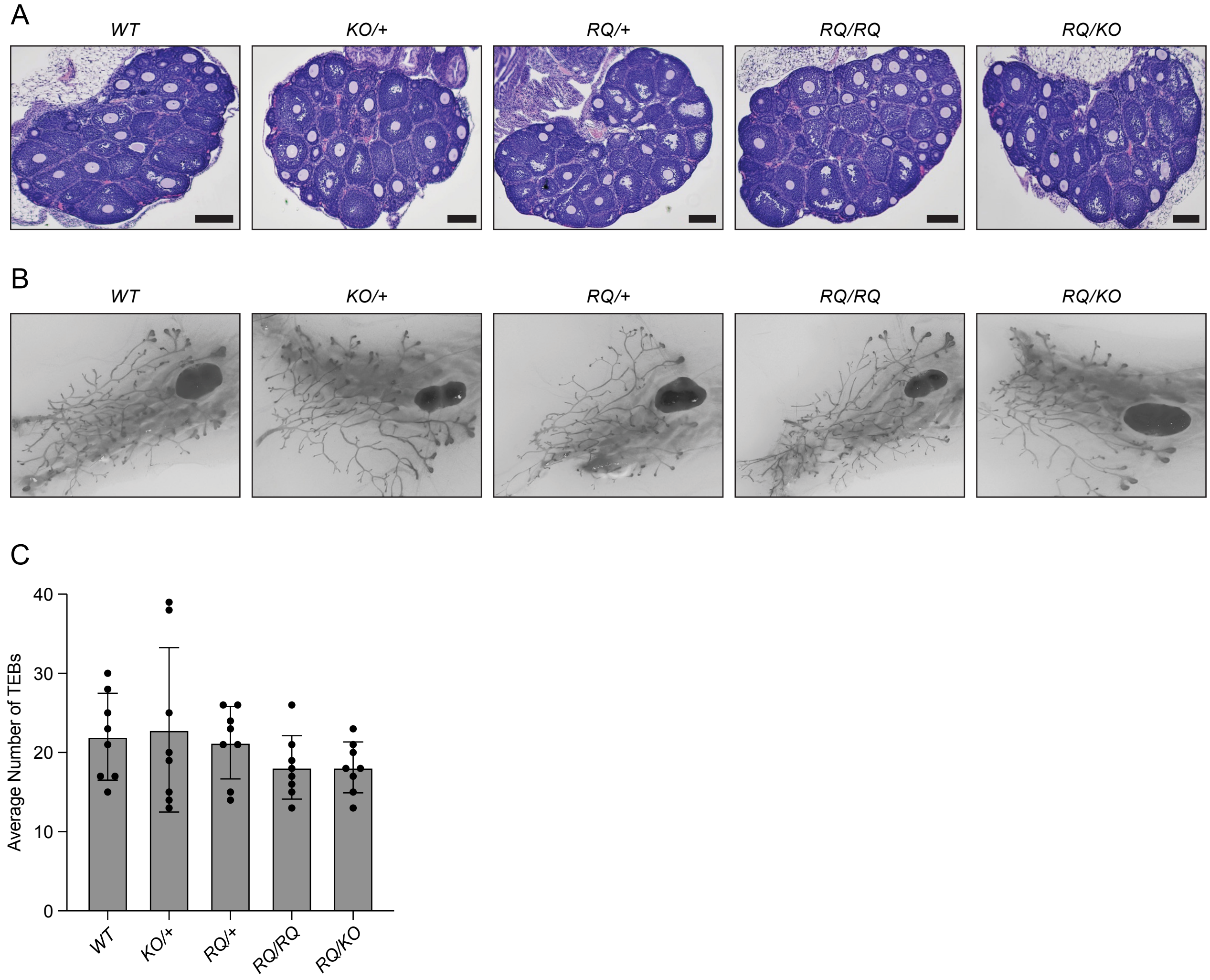

Supplementary Figure 3: Evaluation of Radiation induced RAD51 foci in adult fibroblasts and protection of stalled replication forks in MEFs

A) Representative images of immunofluorescence in adult fibroblasts showing RAD51 foci formation after 3hr of 10Gy IR. Nuclei are marked with DAPI and DSBs with γH2AX. B) Quantification of RAD51 positive nuclei per γH2AX positive of different genotypes. *RQ/KO* MEFs exhibit significantly lower number of RAD51 foci positive nuclei (n=3 biological replicate, Ordinary one-way ANOVA, error bar- SE of mean, **p<0.01). C) Scatter plot showing number of RAD51 foci per nucleus in each genotype (n>100 nuclei per genotype, error bar- SD of mean). D) Scatter plot showing ratio of ldU:CldU (green:red) DNA fibers of MEFs of indicated genotypes after replication forks were stalled by 4mM HU treatment for 3hrs. *Brca1del11* MEFs were used as control for unprotected forks (n=200-300 fibers, error bar- SD of mean).

Supplementary Figure 3

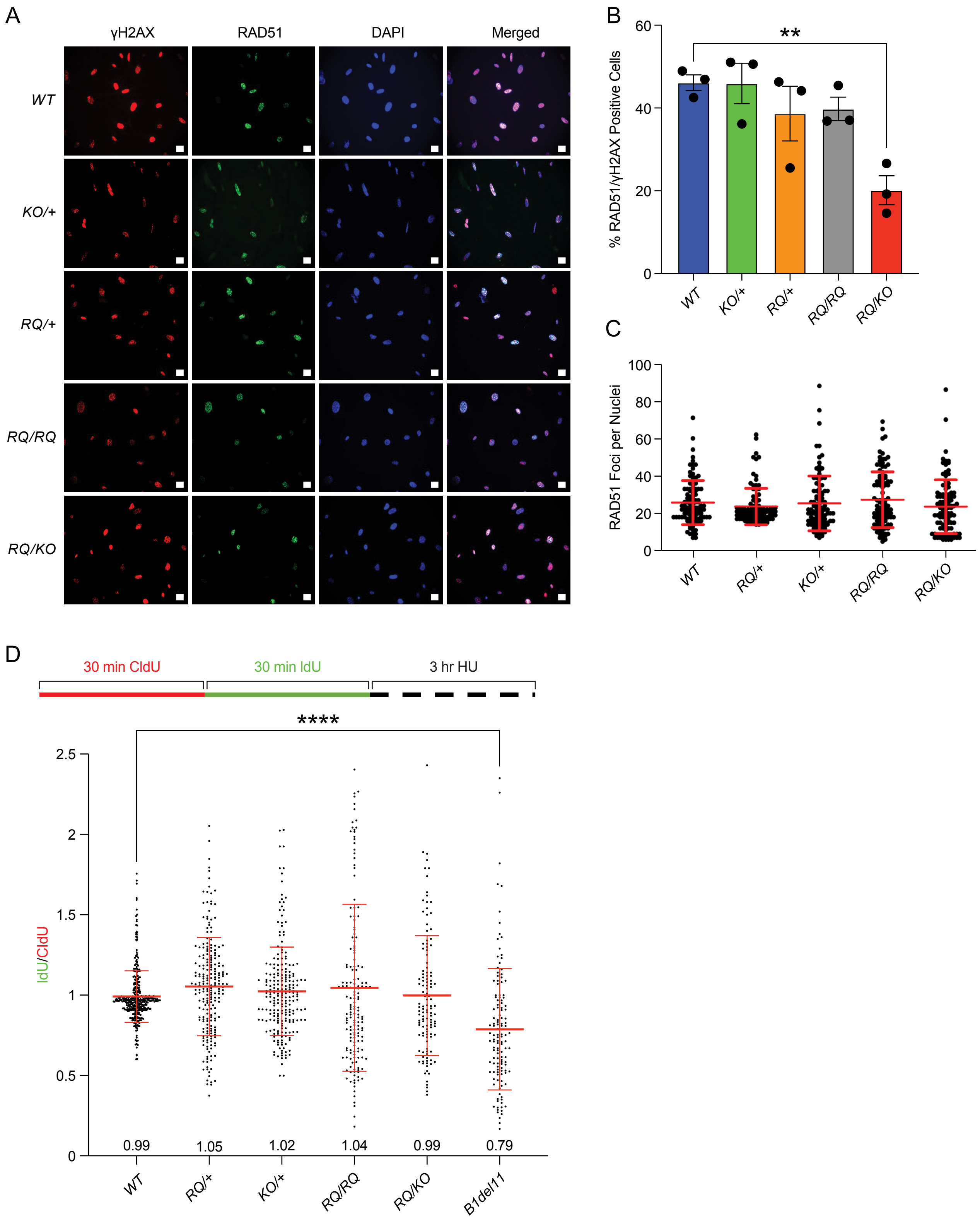

Supplementary Figure 4: Complete blood count-

Peripheral blood was collected from the orbital sinus of 8-week-old male mice of all genotypes and complete blood count was performed (n=3 biological replicates)

Supplementary Figure 4

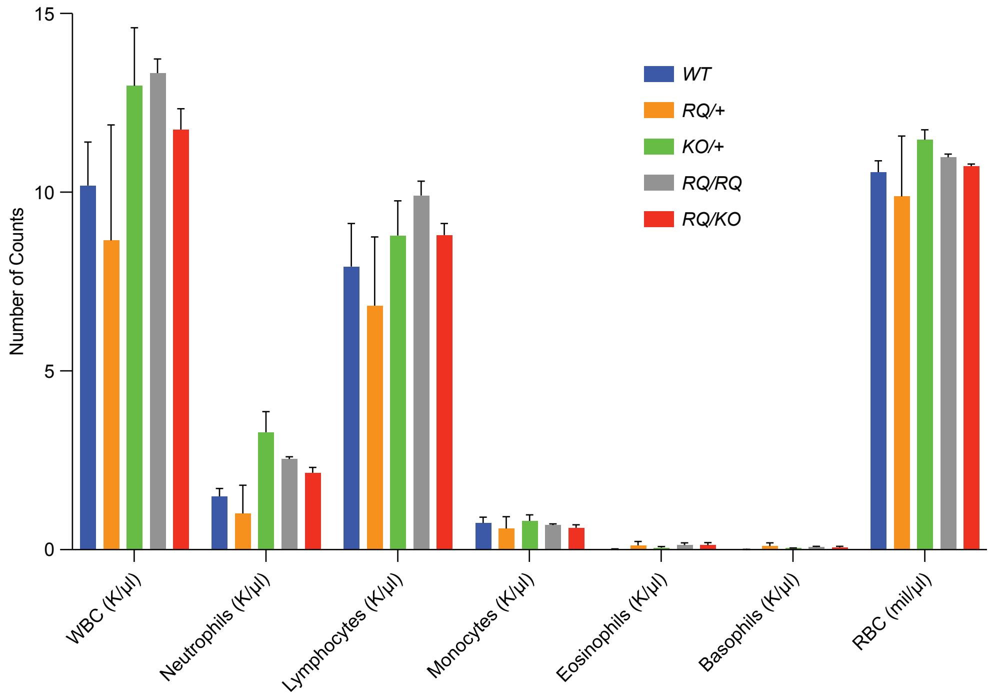

Supp Table 1-primers used for genotyping

**Sequence**	**Amplicon size**	**Application**
RQ Fwd- ctagccagggctgttcagag<br>RQ Rev- cagccaagatgctggagact	WT- 416bp<br>Mutant-520bp	Genotyping for R2971Q allele
Hprt fwd- acagcatctaagaagttttgttctgtcctgg<br>E11 R- ctcaacagagtaggttctttgg	WT- no band<br>Mutant-450bp	Genotyping for *Brca2* KO allele
